# Beyond the Lungs: ICU Oral Care and Gut Resilience—A Hypothesis-Generating Narrative Review

**DOI:** 10.3390/microorganisms14081772

**Published:** 2026-08-12

**Authors:** Tingting Jin, Xueqiang Sun, Xing Zhao, Jiancheng Zhang

**Affiliations:** 1 Department of Critical Care Medicine, Union Hospital, Tongji Medical College, Huazhong University of Science and Technology, 1277 Jiefang Avenue, Wuhan 430022, China; 2 Key Laboratory of Anesthesiology and Resuscitation, Huazhong University of Science and Technology, Ministry of Education, Wuhan 430048, China; 3 Hubei JiangXia Laboratory, Wuhan 430200, China; 4 Department of Critical Care Medicine, The First Affiliated Hospital of Shihezi University, Shihezi 832008, China

**Keywords:** oral–gut microbiome axis, mechanical ventilation, proton pump inhibitors, selective oropharyngeal decontamination, critical care

## Abstract

The oral and gut microbiomes form an interconnected ecosystem that may influence host defense. In the intensive care unit (ICU), endotracheal intubation, broad-spectrum antibiotics, and proton pump inhibitors can disrupt physiological barriers and may facilitate ectopic gut colonization by oral pathobionts. Such translocation could contribute to intestinal inflammation, barrier dysfunction, and systemic immune dysregulation; however, direct clinical evidence in critically ill patients remains limited and largely associative, and much of the mechanistic evidence derives from animal models or non-ICU populations. This hypothesis-generating narrative review synthesizes evidence across oral microbiology, gastroenterology, immunology, and critical care, while explicitly distinguishing clinically observed associations from preclinical mechanisms and proposed ICU pathways. Current evidence supports mechanical toothbrushing as a core component of oral care, whereas routine non-selective chlorhexidine use warrants caution. Saline irrigation and selective oropharyngeal decontamination may be considered in context, but evidence for protecting the gut through oral care remains insufficient for definitive practice recommendations. We also examine probiotics, prebiotics, postbiotics, and fecal microbiota transplantation as investigational approaches and propose standardized prospective multicenter studies to test the oral–gut axis framework.

## 1. Introduction

The human body is an integrated ecosystem where microbial communities maintain health and prevent disease. The oral cavity and gastrointestinal tract host the body’s most complex microbial reservoirs, in continuous dialogue with host physiology. The digestive system mediates the interface between internal and external environments, performing nutrient assimilation and barrier defense [1].

Anatomically contiguous, the mouth and gut form the oral–gut microbiome axis, a sophisticated network involving microbial translocation, components, metabolites, and immune–metabolic signaling [2,3]. The oral cavity, as entry point to respiratory and digestive tracts with rich vascular drainage, influences distal colonization and systemic dissemination [2,4,5,6,7,8]. Growing evidence links oral dysbiosis to systemic diseases including atherosclerosis, rheumatoid arthritis, adverse pregnancy outcomes, liver diseases, stroke, and cardiometabolic and neurodegenerative disorders [2,3,4,5,6,7,8]. Periodontal pathogens like *Porphyromonas gingivalis*, *Fusobacterium nucleatum*, and *Tannerella forsythia* are strongly associated with extra-oral conditions, often via the oral–gut axis [3,9,10]. In the intensive care unit (ICU), critically ill patients experience a concerted assault on microbial homeostasis from endotracheal intubation, broad-spectrum antibiotics, and proton pump inhibitors (PPIs). These interventions may dismantle barriers, induce dysbiosis [11], and facilitate ectopic colonization of oral bacteria in the gut [12,13,14,15,16,17,18,19,20,21,22,23,24,25,26,27,28,29,30,31,32,33,34,35]. This process is hypothesized to actively drive intestinal inflammation, barrier dysfunction, and systemic immune dysregulation, pathways central to ventilator-associated pneumonia (VAP), enterogenic sepsis, and multiple organ dysfunction [36,37,38,39,40].

However, it is important to note that direct evidence linking oral–gut axis disruption to clinical outcomes in critically ill patients remains limited and largely associative. Most mechanistic insights derive from studies of periodontitis, inflammatory bowel disease (IBD), colorectal cancer, animal models, or healthy volunteers, and direct longitudinal evidence from ICU cohorts with paired oral and gut sampling is scarce. These gaps underscore the need for a critical synthesis of existing cross-disciplinary evidence to inform a testable framework for future ICU research.

Accordingly, this hypothesis-generating narrative review has two objectives. First, it synthesizes evidence from oral microbiology, gastroenterology, immunology, and critical care into a testable framework for ICU-associated disruption of the oral–gut axis. Second, it critically appraises oral-care and microbiome-directed strategies without presenting them as definitive clinical guidance. We distinguish established clinical observations from preclinical mechanisms and speculative ICU pathways, identify major confounders and sources of heterogeneity, and define priorities for prospective validation.

## 2. Literature Search and Evidence Synthesis

We conducted a structured narrative search of PubMed and the Medical Literature Analysis and Retrieval System Online (MEDLINE) and supplemented it by backward and forward citation tracking of key reviews and primary studies. Searches covered database inception to 4 August 2026. Search terms addressed the oral and gut microbiomes, oral–gut translocation, critical illness, ICU-related exposures, oral-care interventions, and microbiome-directed therapies. English-language human studies, systematic reviews, randomized trials, observational studies, and mechanistically informative animal or ex vivo studies were eligible when they addressed at least one component of the proposed framework. We prioritized systematic reviews and randomized trials for clinical interventions, longitudinal or strain-resolved human studies for translocation, and primary mechanistic studies when ICU-specific evidence was unavailable.

The final synthesis included 139 cited publications. Records were excluded if they were unrelated to oral–gut connectivity or critical-care interventions, duplicated evidence already represented by a higher-quality or more recent source, lacked sufficient methodological detail, or made mechanistic claims without relevant supporting data. Because this was a hypothesis-generating narrative review rather than a systematic review, study screening was iterative. No Preferred Reporting Items for Systematic Reviews and Meta-Analyses (PRISMA)-style numerical exclusion log was maintained. This approach limits reproducibility and creates a risk of selection bias. When findings conflicted, we gave greater weight to systematic reviews, randomized trials, larger prospective cohorts, and studies with direct paired oral–gut sampling, while retaining discordant findings to characterize uncertainty. Retracted publications were excluded.

For interpretation, we used four evidence categories: (i) established clinical evidence from randomized trials or consistent systematic reviews; (ii) human ICU evidence from longitudinal or observational studies; (iii) human non-ICU evidence; and (iv) preclinical or hypothesis-level evidence. These categories inform the language used throughout: ‘is associated with’ for observational findings, ‘may’ or ‘could’ for biologically plausible extrapolations, and ‘hypothesized’ for pathways not directly validated in ICU cohorts.

## 3. The Oral–Gut Microbiome Axis: Fundamentals

### 3.1. The Distinct Yet Interdependent Ecosystems

A nuanced understanding of the oral–gut axis begins with appreciating the unique environments it connects. The intestinal microbiota is arguably the body’s most complex microbial ecosystem, comprising trillions of bacteria, archaea, fungi, and viruses. Its assembly and function are shaped by a lifelong interplay of host genetics and modifiable environmental factors. These include diet, particularly the intake of fiber, fats, and refined sugars, antibiotic exposure, lifestyle elements like sleep and stress, and broader influences such as urbanization and hygiene practices [41,42]. This community is metabolically and immunologically indispensable. It is pivotal for the education and regulation of the host immune system, the maintenance of intestinal epithelial barrier integrity, the synthesis of essential vitamins (e.g., vitamin K, B vitamins), and the provision of colonization resistance. This latter function, a cornerstone of host defense, is achieved through mechanisms like nutrient competition, spatial occupation, and the production of antimicrobial substances such as short-chain fatty acids (SCFAs) [43]. Disruption of this delicate equilibrium, termed gut dysbiosis, is not merely associative but is increasingly understood to be causally implicated in a vast spectrum of chronic conditions. These conditions include IBD, irritable bowel syndrome, colorectal cancer, obesity, and type 2 diabetes. Associations have also been reported for cardiovascular, neurodegenerative, and respiratory diseases [44,45,46].

In parallel, the oral cavity harbors an exceptionally diverse and dense microbiome, second in scale only to the gut. It is home to over 770 identified bacterial species, which are not randomly distributed but are organized into distinct biogeographical niches shaped by local variations in oxygen tension, pH, salivary flow, and surface topography [47,48,49]. These niches include the buccal mucosa, keratinized gingiva, saliva, tongue dorsum, and highly structured dental-plaque biofilms. Subgingival plaque is an important reservoir for periodontal pathogens such as *Porphyromonas gingivalis* and *Fusobacterium nucleatum* [48,50]. The oral microbiome is inherently dynamic, constantly reshaped by substrate availability from diet, oral hygiene behaviors, habits like smoking, medication use (notably antibiotics and antiseptics), and the host’s local and systemic immune responses. Its composition is dominated by members of the phyla Firmicutes, Proteobacteria, and Bacteroidetes, with key genera including *Streptococcus*, *Neisseria*, *Porphyromonas*, and *Fusobacterium* [51].

### 3.2. The Physiological Barrier and Basal Microbial Exchange

Despite their direct anatomical connection via the digestive tract, a conduit for the daily swallowing of approximately 1011 oral bacteria via saliva [52], the oral and gut microbiomes maintain distinct profiles in health. This separation is enforced by gastric acid, with a pH typically between 1.5 and 3.5, which acts as a sterilizing filter for the vast majority of ingested microbes [53]. Bile acids add another layer of chemical defense, with some possessing direct antibacterial properties through membrane disruption [53]. Within the intestinal lumen, a chemical and immunological shield comprising antimicrobial peptides (e.g., α-defensins), secretory immunoglobulin A (IgA), and the mucous gel layer prevents pathogen adhesion and proliferation [54]. Perhaps the most sophisticated component is biological: the resident gut microbiota itself provides colonization resistance. By outcompeting incoming microbes for nutrients and adhesion sites and by producing inhibitory metabolites such as SCFAs, it effectively guards its territory [55,56]. Consequently, in healthy individuals, most swallowed oral bacteria are eliminated, and survivors typically fail to establish stable, expanding populations, rendering them nearly undetectable in standard fecal microbiome analyses [57].

However, this separation is not absolute, and a basal level of exchange occurs. Strain-level metagenomic analysis of paired saliva and fecal samples showed that oral bacteria can reach the lower gastrointestinal tract. Under permissive conditions, some strains establish low-level colonization, although substantial inter-individual variability exists. Commonly transferred taxa include genera highly prevalent in the mouth, such as *Streptococcus*, *Veillonella*, *Actinomyces*, and *Haemophilus* [58]. This suggests the oral–gut axis facilitates a low-level, natural process of microbial exchange, as further evidenced by the regular detection of salivary *Prevotella* species in stool [59]. Under homeostasis, this exchange is likely inconsequential or even potentially functional. The critical shift occurs when barrier function is compromised, transforming this conduit into a pathway for disease.

### 3.3. Pathological Translocation: When Oral Commensals Become Gut Pathobionts

The collapse of the oral–intestinal barrier escalates microbial exchange from a background process to a central driver of pathology. Periodontitis, a chronic inflammatory oral disease, is a key instigator. Salivary microbiota associated with periodontitis, including members of the Porphyromonadaceae family, *Tannerella*, and *Treponema*, can be transferred to the gut, where they induce intestinal flora imbalance and inflammation [60]. This pathological translocation is markedly amplified in specific disease states. In patients with colorectal cancer, rheumatoid arthritis, and IBD, deoxyribonucleic acid (DNA) and viable cells of specific oral commensals are found enriched in intestinal tissues and tumor microenvironments. *Fusobacterium nucleatum*, *Campylobacter rectus*, and *Peptostreptococcus stomatis* are prominent examples, and their presence frequently correlates with increased disease severity and poorer clinical outcomes [58,61].

**Figure 1:**
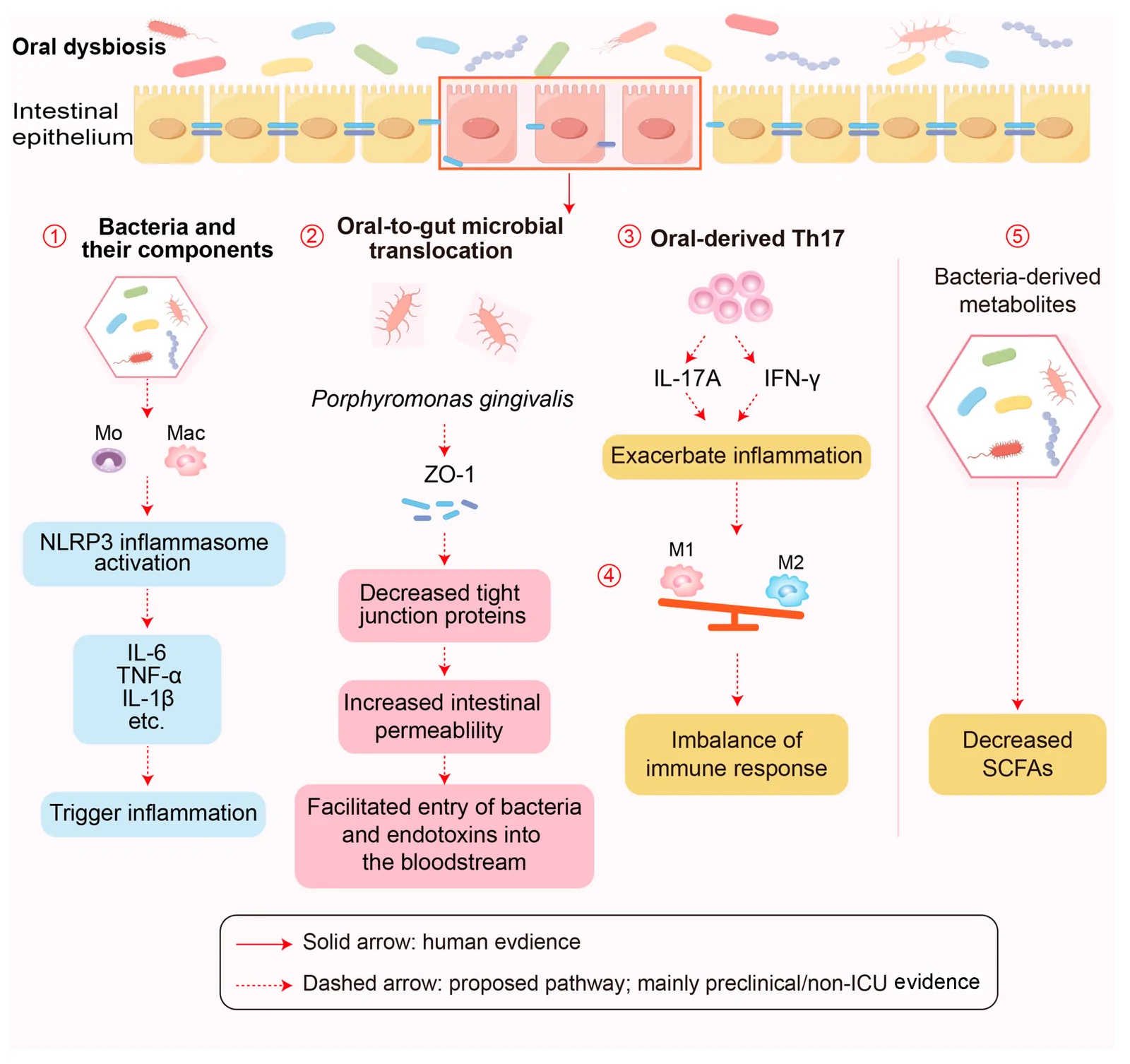
Proposed disruption of the oral–gut microbiome axis by ICU interventions. Endotracheal intubation, broad-spectrum antibiotics, and proton pump inhibitors can alter oral or intestinal microbial ecology. Solid arrows denote relationships supported by human evidence; dashed arrows denote proposed pathways supported mainly by preclinical or non-ICU evidence that require prospective ICU validation. Th17, T-helper 17 cell; Mo, monocyte; Mac, macrophage; DC, dendritic cell; CD8^+^ T, CD8-positive T cell; IL, interleukin; TNF, tumor necrosis factor. The figure was conceived by the authors and was not adapted from a previously published figure.

The mechanisms by which these translocated oral bacteria exert harm are diverse and impactful (Figure 1). Intestinal inflammation, as seen in Crohn’s disease, alters the gut environment in ways that favor colonization by oral bacteria like *Veillonella*. Once established, it can suppress the expression of the apical sodium-dependent bile acid transporter, leading to abnormal luminal accumulation of bile acids that subsequently promote *Clostridium difficile* infection [62]. Hepatic dysfunction can dysregulate bile acid synthesis, depleting intestinal levels of antibacterial bile acids such as 12-ketolithocholic acid (12-KetoLCA). This weakening of the chemical barrier undermines colonization resistance and permits pro-inflammatory, oral-derived *Streptococcus* species to expand ectopically in the intestine [63]. In murine models, *Fusobacterium nucleatum* can aggravate ulcerative colitis through the pro-inflammatory interleukin-17F (IL-17F)/nuclear factor kappa B (NF-κB) signaling cascade. This response increases the expression of interleukin-1 beta (IL-1β), interleukin-6 (IL-6), IL-17F, and tumor necrosis factor alpha (TNF-α) [61,64].

*Porphyromonas gingivalis* serves as a paradigm for an oral pathobiont with systemic reach. Upon reaching the intestine, it directly assaults the epithelial barrier by downregulating the expression of critical tight junction proteins such as zonula occludens-1 (ZO-1), occludin, and tight junction protein 1 (TJP1), thereby increasing gut permeability [65]. It also elevates systemic levels of lipopolysaccharide (LPS), which drives the upregulation of hepatic flavin-containing monooxygenase 3 (FMO3), increases plasma concentrations of the pro-atherogenic metabolite trimethylamine N-oxide (TMAO), and induces both gut dysbiosis and mucosal inflammation [66]. Notably, *Porphyromonas gingivalis* has been detected in extra-intestinal pathological sites like atherosclerotic plaques and pancreatic tumors, powerfully illustrating the systemic dissemination potential of orally derived microbes [61,67,68,69,70,71]. Importantly, this relationship is bidirectional; inflammatory intestinal conditions such as IBD can themselves exacerbate oral microbial dysbiosis, creating a self-perpetuating vicious cycle of inflammation [72]. It is critical to note that most of these mechanistic insights derive from preclinical animal models or observational studies in non-ICU populations; direct evidence in critically ill patients is currently lacking.

### 3.4. Bacterial Adaptations and the Immune-Mediated “Oral–Immune–Gut Axis”

Certain oral bacteria possess inherent traits that enhance their survival during gastrointestinal transit. Acid tolerance is paramount. *Streptococcus mutans*, a primary etiological agent of dental caries, is remarkably aciduric, maintaining viability at pH levels as low as 4.0, which aids its survival through the stomach [73]. Similarly, *Porphyromonas gingivalis* exhibits acid-neutralizing activity via deamination of amino acids into ammonia, which may partially explain its survival during gastric transit and its ability to reach the intestine [74]. This trait becomes clinically significant when gastric acidity is pharmacologically reduced, such as during PPI therapy. Other bacteria exhibit metabolic flexibility. For instance, *Veillonella parvula* can shift its metabolic strategy from fermentation to anaerobic respiration during intestinal inflammation, utilizing amino acids and peptides as carbon sources to facilitate successful colonization [75].

Beyond the direct effects of translocated bacteria, periodontitis may aggravate gut inflammation through an oral–immune–gut axis. Oral infection can alter intestinal immune homeostasis, macrophage polarization, and microbial metabolic profiles. These changes may exacerbate gut dysbiosis and systemic inflammation [76]. Oral pathobionts like *Klebsiella* species can, upon ectopic gut colonization, activate inflammasome pathways and induce IL-1β, which in turn promotes the accumulation of pro-inflammatory T-helper 17 (Th17) cells [77].

The migration and pathogenicity of immune cells are central to this axis. Research using advanced imaging has revealed unexpectedly broad leukocyte trafficking between the gut and other sites [78]. Within the gut, the interplay between periodontal inflammation and intestinal immunity is complex, with Th17 cells playing a crucial role [79]. These Th17 cells can give rise to T helper 1-like (Th1-like) cells that are required for the pathogenesis of colitis [80], a process driven by cytokines such as interleukin-23 (IL-23) [81]. A seminal study elegantly delineated a precise cellular mechanism linking oral and gut inflammation [77]. Periodontitis leads to an increased oral abundance of pathobionts like *Klebsiella* and *Enterobacter*. When swallowed, these bacteria can reach the gut and activate colonic mononuclear phagocytes through the NOD-, LRR- and pyrin domain-containing protein 3 (NLRP3) inflammasome. Concurrently, the oral infection drives the differentiation of pathogen-specific Th17 effector memory (TEM) cells in the cervical lymph nodes. These cells enter the systemic circulation, home to the intestinal lamina propria, and upon re-encountering their cognate oral bacterial antigens, become reactivated. They then secrete potent pro-inflammatory cytokines such as interleukin-17A (IL-17A) and interferon gamma (IFN-γ), causing localized mucosal damage. Crucially, these T cells do not react to the resident commensal gut flora, establishing a direct, antigen-specific immunological link between a remote oral inflammatory site and intestinal pathology. *Fusobacterium nucleatum* can modulate intestinal Th17 responses through the free fatty acid receptor 2 (FFAR2), thereby promoting its colonization [82]. Orally administered *Porphyromonas gingivalis* can also be sampled by microfold (M) cells in Peyer’s patches. Subsequent antigen presentation by dendritic cells may promote local Th17 differentiation and systemic migration [83]. Furthermore, specific oral bacteria like *Prevotella intermedia* can synergistically exacerbate inflammation induced by other oral streptococci, highlighting complex inter-bacterial interactions [84].

It is important to emphasize that the “oral–immune–gut axis” remains a largely theoretical framework in the context of critical illness, derived primarily from animal studies and ex vivo human samples. Direct evidence in mechanically ventilated ICU patients is currently lacking.

## 4. ICU-Induced Disruption of the Oral–Gut Axis

**Figure 2:**
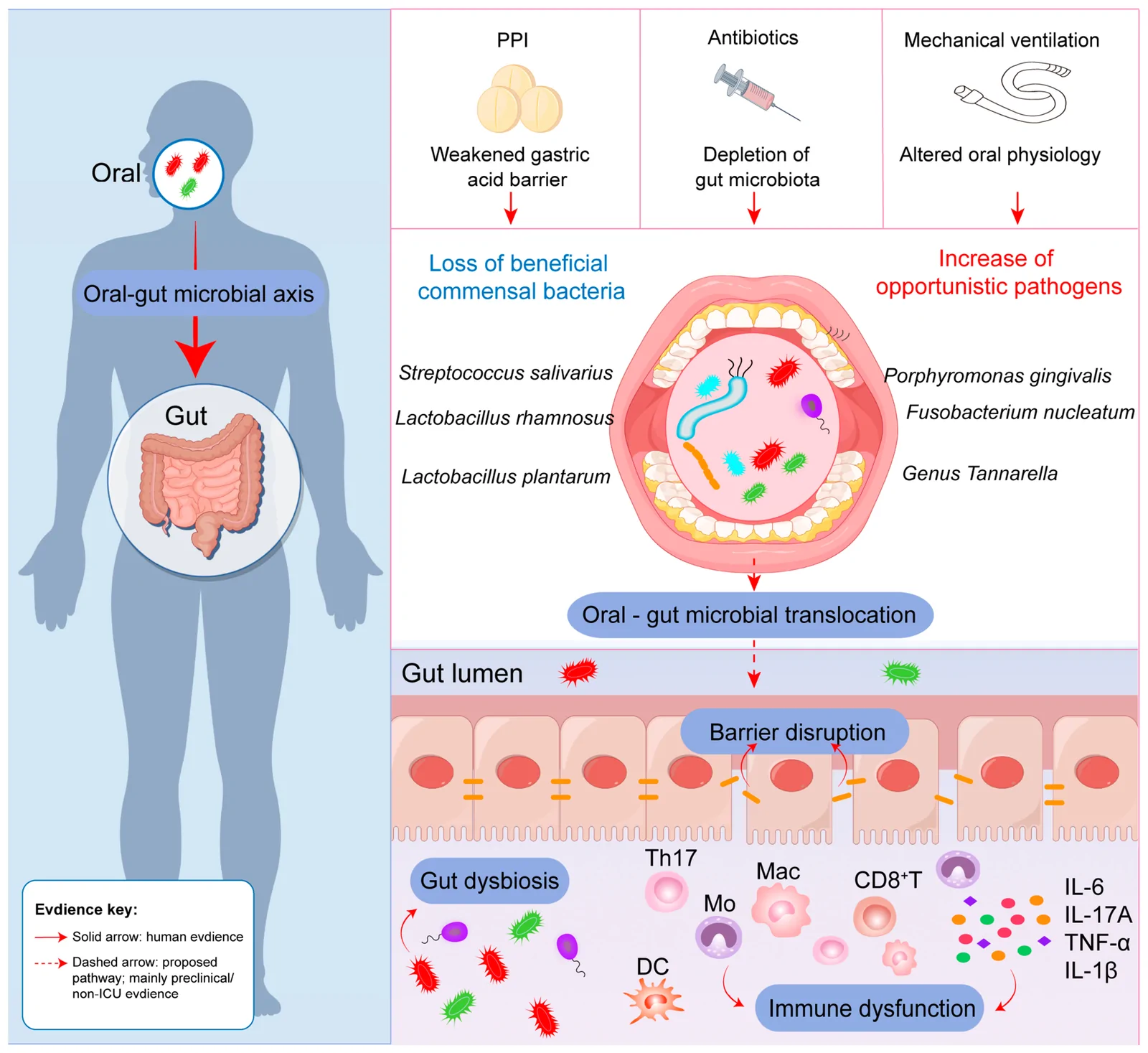
Candidate mechanisms of gut colonization and inflammation by oral pathobionts. The proposed mechanisms include inflammasome activation; disruption of epithelial tight-junction proteins; expansion of Th17 responses; altered M1/M2 macrophage polarization; and changes in microbial metabolites. Solid arrows denote human evidence; dashed arrows denote proposed pathways supported mainly by preclinical or non-ICU evidence. NLRP3, NOD-, LRR- and pyrin domain-containing protein 3; ZO-1, zonula occludens-1; Th17, T-helper 17 cell; Mo, monocyte; Mac, macrophage; IL, interleukin; IFN-γ, interferon-gamma; TNF, tumor necrosis factor; SCFAs, short-chain fatty acids. The figure was conceived by the authors and was not adapted from a previously published figure.

The critically ill patient represents the epitome of a host in which the homeostatic mechanisms guarding the oral–gut axis are overwhelmed. A confluence of severe illness and iatrogenic interventions creates a perfect storm for catastrophic dysbiosis and its sequelae (Figure 2 and Table 1).

### 4.1. Endotracheal Intubation: Engineering a Dysbiotic Oral Reservoir

Endotracheal intubation, while lifesaving for airway management and mechanical ventilation, catastrophically disrupts normal oropharyngeal physiology. Endotracheal intubation profoundly disrupts normal oropharyngeal physiology; the physical presence of the tube, coupled with sedation and impaired consciousness, suppresses speech, mastication, and swallowing, thereby compromising the natural self-cleaning mechanisms of the oral cavity [85]. These actions are not trivial; they facilitate the mechanical clearance of food debris and microbes, while swallowing stimulates salivary flow. Saliva itself is a critical defense fluid, delivering a cocktail of antimicrobial peptides and proteins, including lysozyme, lactoferrin, and IgA, along with buffers and enzymes that collectively provide broad-spectrum protection against invading microbes [95]. Intubated patients frequently experience significant reductions in salivary secretion (xerostomia) due to diminished pharyngeal stimulation, systemic dehydration, and the pharmacological effects of sedatives, opioids, and anticholinergic drugs. The resulting stagnant and moist environment may contain nutrient-rich enteral-feeding residues. These conditions promote microbial overgrowth and biofilm formation on teeth, mucosal surfaces, and the endotracheal tube [86,87]. This ecological shift favors ICU pathogens, including *Staphylococcus aureus* and *Pseudomonas aeruginosa* [88,89].

Although causality has not been definitively established in longitudinal ICU cohorts, cross-sectional and before–after studies consistently demonstrate these associations. A 2025 single-center longitudinal study provided direct, dynamic evidence of this shift, showing that high oral bacterial load at admission was independently associated with the subsequent development of VAP and delirium [90]. A small single-arm study evaluated mechanically ventilated patients receiving standardized oral care. Tongue bacterial load remained high, and microbial α-diversity was significantly lower after extubation than before intubation. These findings indicate loss of diversity and a shift toward a dysbiotic community enriched in opportunistic pathogens [15]. The observation of partial microbial recovery after extubation suggests the process is dynamic and at least partly driven by the presence of the tube and associated physiological impairments. A critical limitation of such studies, however, is their frequent reliance on sampling accessible sites like the tongue. This approach may grossly underestimate dysbiosis in high-risk, biofilm-dense niches like subgingival plaque pockets, which are major reservoirs for pathogens like *Porphyromonas gingivalis* and *Fusobacterium nucleatum* and are directly implicated in systemic dissemination [16,61]. Thus, endotracheal intubation fundamentally and rapidly engineers the oral cavity into a persistent, enriched reservoir of pathobionts, primed for both pulmonary aspiration and gastrointestinal translocation.

### 4.2. Broad-Spectrum Antibiotics: Depleting the Gut’s Indigenous Defense Army

Antibiotic therapy is a non-negotiable yet ecologically devastating mainstay of ICU practice. While targeting life-threatening infections, broad-spectrum agents cause collateral damage by indiscriminately decimating the commensal gut microbiota, the very foundation of colonization resistance. The perturbation of the gut microbiome by antibiotics is profound and multi-faceted [18,19]. A crucial refinement in understanding this process is the “two competing explanations” [20,21]. An increased relative abundance of oral taxa is often observed in the fecal microbiome of critically ill or antibiotic-treated patients. However, this pattern does not necessarily indicate numerical expansion of oral bacteria within the gut. Instead, it is largely a mathematical artifact signaling a catastrophic collapse in the density and diversity of the indigenous gut microbiota. A pivotal mouse experiment made this clear: after one week of broad-spectrum antibiotic treatment, total fecal bacterial load plummeted by over 1000-fold, while the absolute abundance of orally derived bacteria remained unchanged. Their relative proportion, therefore, appeared dramatically inflated [20].

This conceptual distinction is vital for accurate interpretation but in no way diminishes the clinical danger. The antibiotic-induced creation of “ecological vacancies” or “niche opportunities” in the gut allows even small, previously insignificant populations of surviving or newly arriving oral bacteria to establish stable colonization in the absence of competition. Normally benign oral commensals like *Klebsiella pneumoniae* and *Enterococcus faecalis* can become dominant gut inhabitants following antibiotic treatment and trigger robust inflammatory responses. Ectopic colonization by these oral strains activates innate immune pathways, including the NLRP3 inflammasome, and can induce pro-inflammatory Th1 cell responses, leading to significant colonic inflammation and tissue damage [22]. Furthermore, reduced microbiota density is associated with altered host metabolic and immune homeostasis [23,24]. Oral *Porphyromonas gingivalis* can exploit this dysregulated, inflamed environment, exacerbating colitis by skewing the gut microbiota and its metabolic output, specifically through disrupting linoleic acid metabolism and unbalancing the Th17/regulatory T-cell (Treg) axis [25]. Interestingly, this depleted, low-diversity state influences the success of microbiome-based therapies. In fecal microbiota transplantation (FMT), recipients with a pre-treatment gut microbiome characterized by low diversity and high relative abundance of oral taxa, a signature of prior antibiotic use, show higher engraftment success of donor microbiota. This suggests that antibiotic-induced ecological voids enhance niche availability, and some oral commensals like *Streptococcus salivarius* may even play a facilitatory role in donor community establishment [26].

### 4.3. PPIs: Disabling the Gastric Acid Gatekeeper

PPIs are among the most frequently prescribed medications in hospitalized patients, routinely administered for stress ulcer prophylaxis in the critically ill. By potently and persistently suppressing gastric acid secretion, they effectively dismantle a primary chemical barrier of the oral–intestinal axis. However, long-term PPI use is associated with multiple adverse outcomes, including increased risk of gastrointestinal infections (e.g., *Clostridioides difficile*), community-acquired pneumonia, nutrient deficiencies, and potentially cardiovascular and renal events [27,28,29]. Large-scale observational microbiome studies provide consistent evidence: PPI use is associated with reduced gut microbial α-diversity and a compositional shift towards a community structure that resembles dysbiotic states. There is a marked increase in facultative anaerobes typically enriched in the upper gastrointestinal tract and oral cavity, such as *Enterococcus*, *Streptococcus*, *Staphylococcus*, and *Escherichia coli* [30,31,32,33,34,94]. Metagenomic sequencing studies have confirmed and detailed this phenomenon, identifying a consistent enrichment of oral commensals, with genera like *Streptococcus* and *Lactobacillus* being particularly prominent in the gut microbiomes of PPI users [32,33]. These effects appear to be a class-wide, dose-dependent phenomenon [34].

A well-designed 2024 randomized controlled trial (RCT) provided direct causal evidence in healthy volunteers, contrasting PPIs with histamine H2-receptor antagonists (H2RAs) [35]. In healthy volunteers, seven days of PPI use significantly increased oral-to-gut microbial transmission, whereas H2RA use did not. PPI exposure also promoted intestinal colonization by *Fusobacterium nucleatum* and *Streptococcus anginosus*. While these findings in healthy subjects cannot be directly extrapolated to the complex physiological milieu of critically ill patients, they provide compelling proof-of-concept evidence for the acid barrier hypothesis. Crucially, the composition of the oral microbiome itself was not altered by PPI therapy, pinpointing the weakened gastric acid barrier, not an oral source shift, as the primary culprit. In vitro validation confirmed that *Streptococcus anginosus* could not survive at a pH below 5.0, directly linking its survival through the stomach to pharmacological acid suppression. An instructive secondary finding was that the concurrent use of chlorhexidine mouthwash reduced the PPI-driven rise in gut *Streptococcus anginosus*, demonstrating that interventions targeting the oral reservoir can modulate downstream consequences in the gut. The combined impact of antibiotics and PPIs on superinfection risk and prolonged ventilation has been highlighted in an observational study [96], underscoring the clinical relevance of this synergistic effect.

### 4.4. The Synergistic Cascade: A Trajectory of Escalating Dysbiosis

The clinical reality in the ICU is that these three factors rarely act in isolation; they interact synergistically and sequentially, creating an escalatory cascade of dysbiosis.

Step 1: Reservoir Creation. Endotracheal intubation and impaired consciousness promote oral dysbiosis and the overgrowth of pathogens, creating a loaded “gun”.

Step 2: Gate Opening. Concurrent PPI use reduces gastric acidity, disabling a key safety mechanism and allowing these acid-sensitive oral pathobionts to survive transit, effectively “pulling the trigger”.

Step 3: Ground Preparation. Broad-spectrum antibiotic therapy devastates the indigenous gut microbiota, destroying colonization resistance and creating vacant ecological niches in the intestine, “preparing the battlefield”. The translocated oral bacteria then colonize the vulnerable gut ecosystem, where they can disrupt barrier function, activate local and systemic immune pathways, and fuel inflammation. Emerging human evidence directly supports this translocation cascade in critically ill patients. A 2024 comparative microbiome study identified significant strain sharing between oropharyngeal and rectal swabs from ICU patients. Near death, oropharyngeal networks showed the greatest loss of modularity and enrichment of biofilm-forming pathobionts, including *Streptococcus oralis*, methicillin-resistant *Staphylococcus aureus* (MRSA), and *Enterococcus faecalis* [93]. These findings provide direct evidence that oral dysbiosis and gut colonization are linked in the ICU setting, reinforcing the biological plausibility of the cascade described above. This cascade is a fundamental, underappreciated driver of ICU-acquired complications, conceptually linking routine oral care directly to outcomes far beyond the prevention of pneumonia. Direct evidence for each step in ICU patients is still incomplete and requires prospective validation.

### 4.5. Evidence Hierarchy, Population Heterogeneity, and Alternative Explanations

The evidence supporting the proposed ICU oral–gut axis is uneven. Evidence that intubation alters oral ecology and that antibiotics or acid suppression perturb the gut microbiome is supported by human studies, including randomized evidence for acid suppression in healthy volunteers. In contrast, evidence that oral-derived organisms causally drive intestinal inflammation or systemic organ dysfunction in critically ill patients is derived mainly from animal models, ex vivo systems, and non-ICU disease cohorts. ICU studies showing strain sharing or temporal associations strengthen biological plausibility but do not establish directionality or mediation of clinical outcomes.

Interpretation is further complicated by heterogeneity among medical, surgical, trauma, neurocritical-care, burn, transplant, and other immunocompromised populations. Baseline oral health, illness severity, shock, organ failure, enteral or parenteral nutrition, feeding route, antimicrobial class and duration, acid-suppressive therapy, sedation, bowel motility, comorbidities, and local oral-care protocols can each influence both microbiome composition and outcomes. These factors may confound apparent relationships between oral–gut dysbiosis and complications. Reverse causation is also plausible: severe critical illness may simultaneously cause dysbiosis and adverse outcomes, while treatment intensity may be a marker rather than a cause of microbiome disruption. Future studies should therefore prespecify confounders, measure absolute as well as relative microbial abundance, and use longitudinal designs capable of testing temporal sequence and mediation.

## 5. Reimagining ICU Oral Care: Toward Ecological Stewardship

Given the oral cavity’s demonstrated role as a nexus for systemic infection and inflammation, meticulous oral care is a non-negotiable component of high-quality critical care. Implementation remains challenging because endotracheal and other tubes restrict access. Sedation or delirium may limit patient cooperation, while nursing practices and knowledge vary. Many units also lack standardized, protocol-driven approaches [85,97,98]. A systematic review of practices confirms this variability and underscores the need for evidence-based protocols [99]. The traditional and primary goal of oral care has been the prevention of VAP, justified by the well-established pathogenesis of microaspiration of oropharyngeal secretions into the lower airways [100,101]. Microbial sequencing studies have identified shared bacterial strains in the oral cavity and lungs of patients with VAP [102,103,104]. A high oral bacterial load at ICU admission independently predicted subsequent VAP and delirium in one longitudinal study [90].

The colonization of dental plaque by respiratory pathogens is a key event preceding infection [91,92], and genetic studies confirm the relatedness of isolates from plaque and the lungs in VAP patients [105]. Even commensal oral communities can interfere with the integration of pathogens like *P. aeruginosa* [106], but this balance is lost in dysbiosis. The etiological agents of VAP often originate from this oral reservoir [107], with pathogens such as *Porphyromonas gingivalis* implicated in worsening aspiration pneumonia [108]. Early trials demonstrated that oral care could reduce pneumonia incidence and modify mortality risk [109,110], laying the foundation for modern protocols.

However, in light of the oral–gut axis, the purpose and potential impact of oral care must be expansively redefined. It is not solely a pulmonary protection strategy but a fundamental intervention to protect the integrity of the oral–gut axis and mitigate the systemic inflammatory cascade that can originate from it [36,37,38,39,40].

### 5.1. The Fall of a Former Standard: Re-Evaluating Chlorhexidine

For decades, chlorhexidine gluconate (CHG) mouthwash or gel was the cornerstone of ICU oral care protocols, prized for its broad-spectrum bactericidal and fungicidal activity. This practice was bundled into VAP prevention guidelines worldwide. However, accumulating evidence from high-quality meta-analyses has fundamentally challenged this doctrine. CHG reliably reduces quantitative oral bacterial colonization. However, a 2014 meta-analysis found no significant reduction in VAP among mixed medical–surgical ICU populations. The analysis also identified an association with increased mortality (odds ratio (OR) 1.25, 95% confidence interval (CI) 1.05–1.50) [111,112]. A 2024 network meta-analysis of 16 RCTs reinforced this finding. CHG did not significantly reduce VAP, ICU length of stay, mechanical-ventilation duration, or mortality at any tested concentration [113]. Several meta-analyses reported a mortality signal among patients receiving routine CHG, primarily from observational and secondary analyses [111,114,115,116]. However, a subsequent large cluster-randomized trial did not confirm this signal [117]. The evidence for combining toothbrushing with CHG also requires careful interpretation, as benefits may stem from the mechanical action rather than the antiseptic [118].

The mechanisms underlying this potential for harm are multifactorial and align with principles of microbial ecology. First, mucosal toxicity: Frequent application, particularly of the 2% concentration, is associated with a significant incidence of oral mucosal injury, including ulcerations, erythema, and desquamation [119]. These lesions compromise the epithelial barrier, potentially increasing pain, discouraging adequate care, and facilitating the systemic translocation of bacteria. Second, emerging resistance: There is growing documentation of reduced susceptibility to CHG among relevant ICU pathogens such as *Pseudomonas aeruginosa*, MRSA, and *Enterobacter* species [120,121]. This resistance may develop under the selective pressure of long-term, sub-lethal exposure. Third, and perhaps most critically in the context of the microbiome axis, ecological disruption: CHG’s non-selective, “scorched-earth” antimicrobial approach disrupts the balance of the oral microbiome. By indiscriminately suppressing commensal Streptococci and other early colonizers that contribute to a healthy microbial landscape, it creates ecological vacancies. These vacancies can then be exploited by more virulent, often multidrug-resistant, opportunistic pathogens, potentially paradoxically increasing the risk of subsequent infection. Furthermore, a damaged mucosa combined with an altered microbiome may paradoxically enhance biofilm formation on endotracheal tubes. A multi-center stepped-wedge cluster RCT (the CHORAL trial) found that discontinuing chlorhexidine and implementing a bundled oral care protocol did not worsen mortality or other key outcomes, supporting the safety of de-adopting this agent [117].

CHG may retain a limited role in specific circumstances. Examples include short-term use before invasive procedures or use in ICUs with high multidrug-resistant Gram-negative colonization where selective oropharyngeal decontamination (SOD) is unavailable. The evidence against CHG is strongest for routine, prolonged use in mixed medical–surgical ICUs; its use in selected populations or for limited durations has not been adequately studied.

### 5.2. The Rise of Evidence-Based, Ecological Alternatives

**Figure 3:**
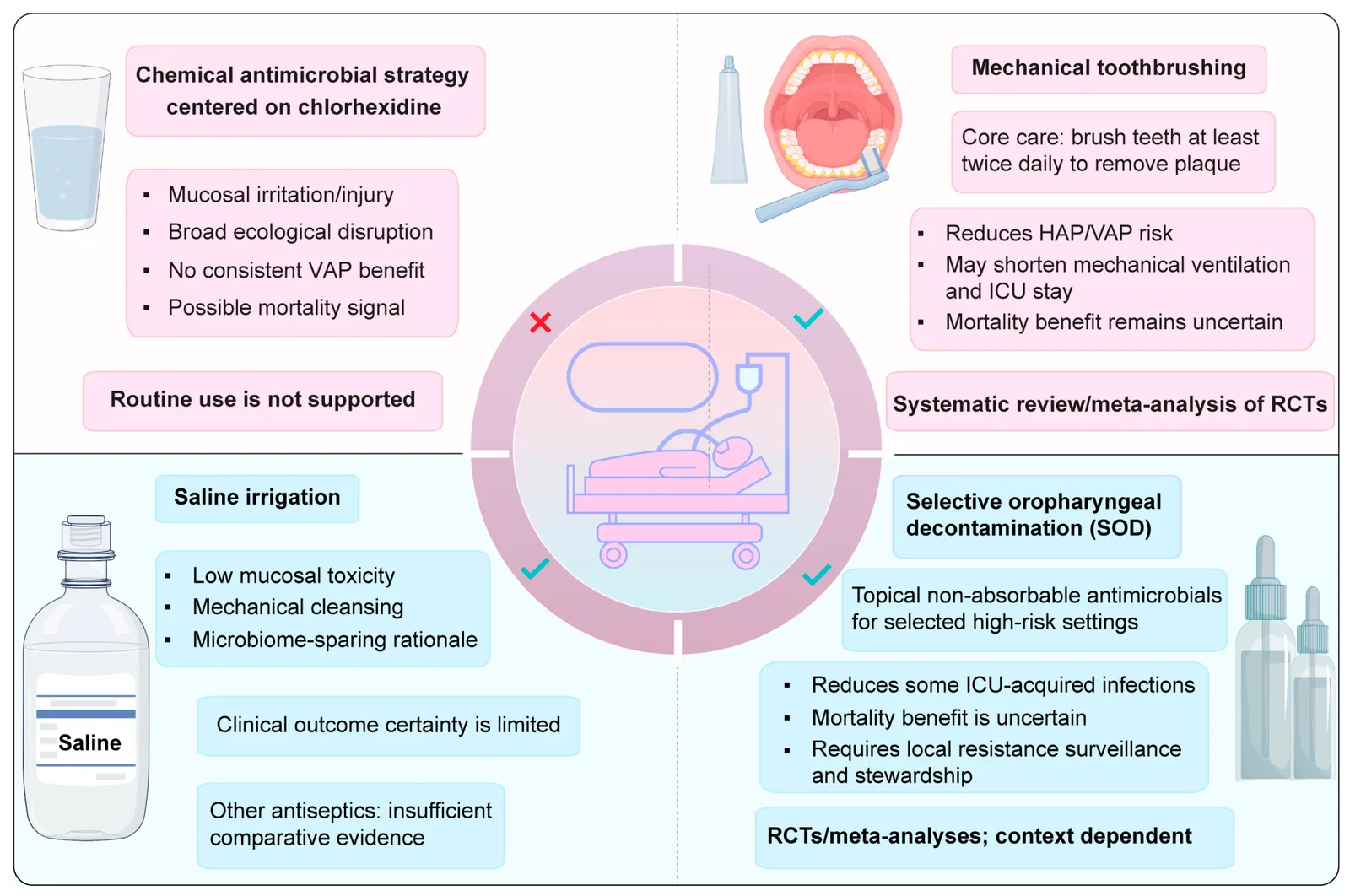
Evidence-informed oral-care strategies for critically ill patients. The figure compares a chlorhexidine-centered chemical antimicrobial strategy with mechanical toothbrushing, saline irrigation, and selective oropharyngeal decontamination. The wording reflects the strength and limitations of the available clinical evidence. The dashed vertical lines are visual separators and do not represent evidence strength or causal relationships. ICU, intensive care unit; HAP, hospital-acquired pneumonia; VAP, ventilator-associated pneumonia; RCT, randomized controlled trial; SOD, selective oropharyngeal decontamination. The figure was conceived by the authors and was not adapted from a previously published figure.

This compelling evidence has catalyzed a paradigm shift in ICU oral care, moving away from reliance on non-selective chemical agents towards strategies that are more physiological, targeted, and focused on mechanical disruption (Figure 3 and Table 2).

#### 5.2.1. Mechanical Debridement: Re-Establishing Tooth Brushing as the Cornerstone

The physical removal of dental plaque biofilm using a soft-bristled toothbrush is now unequivocally recognized as the foundational practice. A landmark 2024 systematic review and meta-analysis encompassing 15 randomized trials and over 10,000 patients provided robust, high-level evidence [122]. The analysis concluded that daily tooth brushing may be associated with the risk of hospital-acquired pneumonia (HAP) and ICU mortality among mechanically ventilated patients. It was also associated with clinically meaningful reductions in the duration of mechanical ventilation (by approximately 1.24 days) and ICU length of stay (by about 1.78 days). Importantly, brushing twice daily versus more frequent intervals was associated with similar effect estimates, suggesting a potential ceiling effect for frequency. This mechanical action directly targets the structured, adherent biofilm reservoir, a sanctuary for pathogens, without causing the collateral ecological damage associated with antiseptics. Reflecting this evidence, guidelines from the Society for Healthcare Epidemiology of America (SHEA) now strongly recommend tooth brushing as a core component of oral care for ventilated patients and explicitly advise against the routine use of chlorhexidine [127].

While this meta-analysis provides high-level evidence for tooth brushing in preventing HAP and reducing ICU mortality, the included trials were predominantly conducted in general medical–surgical ICUs. Generalizability to specialized units (e.g., neurosurgical, burn, immunocompromised patients) requires further study. A recent quasi-experimental interrupted time-series study in eight ICUs evaluated the real-world impact of replacing chlorhexidine with toothbrushing and reinforcing head-of-bed elevation. Although the primary analysis showed a significant decline in VAP incidence, the effect was attenuated and non-significant after adjusting for time-varying covariates, underscoring the feasibility but also the uncertainty of this ecological approach [123]. These findings are further supported by a 2025 RCT in elderly ventilated patients, which found that chlorhexidine significantly reduced oral bacterial diversity but did not improve VAP incidence, ventilation duration, ICU stay, or 28-day mortality compared with saline [134].

#### 5.2.2. Physiological Irrigation: The Re-Emergence of Normal Saline

In response to the concerns surrounding antiseptics, normal saline (0.9% sodium chloride) has re-emerged as a safe and effective irrigation solution. A network meta-analysis compared several oral-care solutions. Antiseptic agents such as chlorhexidine were associated with higher mortality, whereas normal saline was associated with lower ICU mortality (risk ratio 0.18, 95% CI 0.04–0.88) [124]. Saline works primarily through mechanical action: it loosens, dilutes, and flushes away debris, thick secretions, and loosely adherent biofilm. Its advantages are its safety profile—it is isotonic, non-irritating, and poses no risk of mucosal injury or antimicrobial resistance—and its ecological neutrality. It cleanses without attempting to sterilize, thereby preserving the existing microbiome structure. When combined with regular tooth brushing and suctioning, saline-based care offers a gentle, effective, and low-risk approach to maintaining oral hygiene, particularly suitable for patients with fragile oral tissues or where microbiome preservation is a priority [125,126].

#### 5.2.3. SOD: A Paradigm of Precision Targeting

SOD represents a sophisticated “middle path” between non-selective antiseptics and purely mechanical care. It involves the topical application of a paste or gel containing non-absorbable antibiotics (typically polymyxin E/colistin and tobramycin) and an antifungal agent (nystatin) directly to the oropharyngeal mucosa. The philosophy is one of precision: unlike chlorhexidine, SOD aims to selectively suppress known, high-threat pathogenic Gram-negative bacilli (e.g., *Pseudomonas aeruginosa*, *Klebsiella pneumoniae*) and *Candida* species without causing broad disruption to the commensal oral and gut flora [128].

Large-scale meta-analyses of RCTs have demonstrated that SOD and Selective Digestive Decontamination (SDD) are associated with reduced mortality and infection rates among critically ill, mechanically ventilated patients compared to standard care [114,129]. A recent multinational SuDDICU trial provides the largest and most contemporary assessment of SDD’s impact in a broader ICU context [130]. In over 9000 mechanically ventilated patients, SDD did not achieve a statistically significant reduction in the primary endpoint of 90-day in-hospital mortality. However, it delivered significant and clinically important reductions in key secondary infectious outcomes: the incidence of new bloodstream infections (4.9% vs. 6.8%) and the acquisition of new antibiotic-resistant organisms (16.8% vs. 26.8%). This dissociation between mortality and infection endpoints underscores the multifactorial nature of death in critical illness but firmly establishes SDD’s efficacy in mitigating specific complications linked to oral–gut axis disruption. A critical and counterintuitive finding from these long-term studies is that neither SOD nor SDD leads to an increase in the prevalence of antibiotic-resistant Gram-negative bacteria in the ICU. In fact, they are associated with a decreased carriage and infection rate of bacteria resistant to third-generation cephalosporins and carbapenems [128,131,132]. This is likely because by preventing heavy colonization and overgrowth of these pathogens, the interventions reduce the overall bacterial burden and patient-to-patient transmission, offsetting any local selective pressure from the topical agents. A five-year case–control study further reinforced these benefits, showing that SOD implementation was associated with a lower incidence of infections with extended-spectrum β-lactamase (ESBL)-producing *Klebsiella pneumoniae*, as well as reduced rates of VAP and ICU mortality [133]. SOD thus stands as a powerful example of how a localized, microbiome-sparing intervention can yield significant systemic benefits, though its adoption requires careful consideration of local resistance patterns and institutional protocols.

Despite these encouraging findings, the adoption of SOD/SDD remains controversial and geographically variable. Important concerns remain. These include long-term ecological effects on ICU resistance reservoirs and limited efficacy data in settings with high baseline carbapenem resistance. Additional barriers include preparation and administration of topical antibiotic pastes, regulation, and reimbursement. Therefore, SOD/SDD should currently be considered an advanced strategy for ICUs with low baseline resistance and robust antimicrobial stewardship programs, rather than a universal standard.

### 5.3. Microbiome-Directed Adjuncts: Probiotics, Prebiotics, Postbiotics, and FMT

Microbiome-directed therapies could, in principle, complement oral ecological stewardship by restoring colonization resistance, microbial metabolic function, and mucosal immune homeostasis [135,136,137]. Probiotics may competitively exclude pathobionts, reinforce epithelial tight junctions, and modulate inflammatory signaling; prebiotics may preferentially support commensal taxa and SCFA production; and postbiotics may deliver microbial metabolites or structural components without administering live organisms. FMT offers the most direct ecosystem-level replacement and may fill antibiotic-created ecological vacancies. These mechanisms are biologically plausible along the oral–gut axis and are consistent with broader oral–gut microbiota literature [138]. Sex-specific host factors may also influence oral–gut axis integrity and treatment responsiveness, but remain insufficiently investigated in critically ill populations [139].

Nevertheless, these approaches should be considered investigational in critically ill patients. ICU-specific efficacy data are sparse, formulations and endpoints are heterogeneous, and live-biotherapeutic products or FMT may introduce infection-transmission risks in patients with disrupted barriers or profound immunosuppression. Moreover, restoring the gut microbiota would not necessarily eliminate the upstream oral reservoir. Future trials should therefore combine rigorous donor or product screening with strain-resolved longitudinal oral and fecal sampling, antimicrobial-exposure data, safety surveillance, and clinically relevant outcomes. At present, none of these approaches can be recommended specifically to normalize the ICU oral–gut axis outside research protocols.

## 6. Limitations of the Evidence Base and Future Research

The current evidence base has important conceptual and methodological limitations. Direct ICU studies with paired oral and fecal sampling are scarce, most mechanistic evidence comes from animal models or non-ICU populations, and observational studies cannot reliably separate microbiome effects from illness severity or treatment exposure. Differences in 16S rRNA gene sequencing regions, shotgun metagenomic depth, bioinformatic pipelines, taxonomic resolution, absolute versus relative abundance measures, sampling sites, collection timing, storage conditions, and contamination control limit comparability. Oral sampling restricted to saliva or the tongue may miss subgingival biofilm reservoirs, whereas fecal samples may not represent mucosa-associated communities. The narrative design of this review, including iterative study selection without a numerical exclusion log, also permits selection bias.

Future studies should prioritize prospective, longitudinal, multicenter ICU cohorts with predefined serial sampling of multiple oral niches and feces. Collection, storage, sequencing, and bioinformatic protocols should be harmonized, with absolute microbial quantification and strain-resolved metagenomics. Studies should also measure barrier, immunological, metabolomic, antimicrobial-exposure, nutritional, and clinical outcomes in parallel. Analyses should prespecify illness severity, comorbidities, feeding, acid suppression, antimicrobial exposure, and ICU subtype, and should test reverse causation and mediation. Randomized trials of oral-care or microbiome-directed interventions should use standardized safety monitoring and clinically meaningful outcomes such as incident infection, organ-failure-free days, length of stay, and mortality, while prospectively evaluating medical, surgical, trauma, neurocritical-care, burn, and immunocompromised subgroups.

## 7. Conclusions

The oral–gut axis provides a biologically plausible framework linking routine ICU interventions, microbial ecological disruption, and systemic inflammation, but it remains hypothesis-generating rather than clinically validated. Current evidence supports careful, mechanically focused oral care and cautions against assuming that broad antisepsis protects downstream microbial ecosystems. Whether preserving or restoring oral–gut ecological continuity improves outcomes beyond established pulmonary benefits must be tested in rigorously designed prospective ICU studies.

## Figures and Tables

**Table 1:** Factors Affecting the Oral–Gut Microbiome Axis in the ICU and Their Mechanisms.

Factor	Mechanism of Disruption	Clinical Implications	References
Endotracheal Intubation	Suppresses swallowing, speech, and mastication → reduces salivary flow and natural clearance; promotes biofilm formation on teeth, mucosa, and tube; favors overgrowth of pathogens (*S. aureus*, *P. aeruginosa*, *Candida*)	Oral dysbiosis, high bacterial load → increased aspiration, VAP, delirium; facilitates translocation of oral pathobionts to the gut	[15,85,86,87,88,89,90,91,92]
Broad-Spectrum Antibiotics	Depletes commensal gut microbiota (↓ density, ↓ diversity) → collapses colonization resistance; creates ecological niches; alters immune homeostasis (↓ IgA, ↓ Tregs); relative enrichment of oral taxa in feces is largely a mathematical artifact of gut depletion	Gut dysbiosis, loss of barrier function; promotes *C. difficile* infection; enables ectopic colonization by oral *Klebsiella*, *Enterococcus* → Th1/Th17 inflammation, systemic endotoxemia	[18,19,20,21,22,23,24,25,26,93]
Proton Pump Inhibitors (PPIs)	Potently suppress gastric acid (pH ↑) → disable the primary chemical barrier; enhance survival and gastric transit of acid-sensitive oral bacteria (*S. anginosus*, *F. nucleatum*); increase oral-to-gut microbial transmission	Gut microbiome shift (↑ *Streptococcus*, ↑ *Lactobacillus*); increased risk of enteric infections, pneumonia, and systemic inflammation; dose-dependent effect	[27,28,29,30,31,32,33,34,35,94]
Sedation & Immobility	Reduce gastrointestinal motility and cough reflex; impair mucociliary clearance and secretion drainage; promote stasis and bacterial overgrowth in both oral and intestinal compartments	Facilitates microbial translocation, worsens dysbiosis, increases risk of aspiration, VAP, and gut-derived sepsis	[85] (indirect)

Abbreviations: ICU, intensive care unit; VAP, ventilator-associated pneumonia; PPIs, proton pump inhibitors; Tregs, regulatory T cells; IgA, immunoglobulin A. “↑” denotes an increase or elevation, whereas “↓” denotes a decrease or reduction.

**Table 2:** Comparison of Oral Care Strategies in the ICU.

Oral Care Method	Key Features/Mechanism	Clinical Outcomes and Evidence Level	Practical Considerations	References
Chlorhexidine-based Care	Broad-spectrum antiseptic (0.12–2%); reduces total bacterial load via membrane disruption	Systematic reviews/meta-analyses and randomized trials: No significant VAP reduction in mixed ICU; meta-analyses show ↑ mortality (OR 1.25); mucosal injury, resistance selection, ecological disruption	Avoid routine use in general medical–surgical ICUs; may have limited role in short-term high-risk scenarios (not proven)	[111,112,113,114,115,116,117,119,120,121]
Standardized Oral Care without Chlorhexidine	Bundle: oral assessment, tooth brushing, moisture maintenance, secretion suction; no antiseptic	Cluster-randomized and quasi-experimental evidence: No mortality benefit over chlorhexidine, but feasible and safe; CHORAL trial showed de-adoption did not worsen outcomes	Implement as baseline; ensures mechanical cleaning and mucosal protection	[121,122,123]
Saline-based Irrigation	Isotonic 0.9% NaCl; mechanical flushing and dilution of debris and loosely adherent biofilm; preserves microbiome integrity	Network meta-analysis plus mechanistic rationale: Network meta-analysis associated with ↓ ICU mortality (RR 0.18) (primary evidence); no mucosal toxicity or resistance	Ideal for fragile mucosa, renal impairment, or when microbiome preservation is prioritized	[124] (primary), [125,126]
Tooth Brushing	Soft-bristled brush physically disrupts dental plaque and biofilms; removes pathogen reservoir	Systematic review/meta-analysis of randomized trials: Meta-analysis (15 RCTs, >10,000 patients) showed ↓ HAP, ↓ ICU mortality, ↓ MV duration (1.24 d), ↓ ICU LOS (1.78 d). SHEA strongly recommends ≥ twice daily	No additional benefit from more frequent brushing; compatible with saline/SOD; cornerstone of care	[122,123,127]
SOD	Topical non-absorbable paste (polymyxin/tobramycin + nystatin) applied to oropharynx; selectively targets Gram-negatives and *Candida*	Randomized trials and meta-analyses, with context-dependent generalizability: Large RCTs/meta-analyses: ↓ VAP, ↓ bloodstream infections, ↓ acquisition of resistant organisms; SuDDICU trial showed no mortality benefit but significant infection reduction. Does not increase resistance in low/moderate resistance settings	Advanced strategy for high-risk ICUs with low baseline resistance and active stewardship; not a universal standard	[114,128,129,130,131,132,133]
Saliva Substitutes/Moisturizers	Artificial saliva or moisturizing gel; maintains hydration and mucosal integrity; supports endogenous antimicrobial peptides	Physiological rationale; no direct outcome evidence: Prevents xerostomia and mucosal cracking; indirect benefit for barrier function and patient comfort (based on physiological rationale)	Adjunct to brushing/saline; especially useful in sedated or dehydrated patients	[95] (theoretical support)

Abbreviations: ICU, intensive care unit; VAP, ventilator-associated pneumonia; HAP, hospital-acquired pneumonia; SHEA, Society for Healthcare Epidemiology of America; SOD, selective oropharyngeal decontamination; MV, mechanical ventilation; LOS, length of stay; RCT, randomized controlled trial. “↑” denotes an increase or elevation, whereas “↓” denotes a decrease or reduction.

## Data Availability

No new data were created or analyzed in this study. Data sharing is not applicable to this article.

## Abbreviations

The following abbreviations are used in this manuscript:

CHG	Chlorhexidine Gluconate
COPD	Chronic Obstructive Pulmonary Disease
FMT	Fecal Microbiota Transplantation
H2RA	Histamine H2-Receptor Antagonist
HAP	Hospital-Acquired Pneumonia
IBD	Inflammatory Bowel Disease
ICU	Intensive Care Unit
LPS	Lipopolysaccharide
MRSA	Methicillin-Resistant *Staphylococcus aureus*
PPI	Proton Pump Inhibitor
SCFA	Short-Chain Fatty Acid
SDD	Selective Digestive Decontamination
SOD	Selective Oropharyngeal Decontamination
VAP	Ventilator-Associated Pneumonia
